# A-Kinase Anchor Protein 4 (proAKAP4): Protein Molecule–Based Fertility Marker of Indonesian Dairy Bull and Its Correlation With Frozen-Thawed Sperm Quality

**DOI:** 10.1155/vmi/8367714

**Published:** 2025-02-24

**Authors:** Berlin Pandapotan Pardede, Erif Maha Nugraha Setyawan, Syahruddin Said, Asmarani Kusumawati, Bambang Purwantara, Mulyoto Pangestu, Erdogan Memili

**Affiliations:** ^1^Research Center for Applied Zoology, National Research, and Innovation Agency (BRIN), Bogor, West Java, Indonesia; ^2^Department of Reproduction, Obstetrics and Gynecology, Faculty of Veterinary Medicine, Universitas Gadjah Mada, Yogyakarta, Indonesia; ^3^Division of Reproduction and Obstetrics, School of Veterinary Medicine and Biomedical Sciences, IPB University, Bogor, Indonesia; ^4^Department of Obstetrics and Gynecology, School of Clinical Sciences, Faculty of Medicine, Nursing & Health Sciences, Monash University, Victoria, Australia; ^5^Cooperative Agriculture Research Center, College of Agriculture, Food and Natural Resources, Prairie View A&M University, Prairie View, Texas, USA

**Keywords:** dairy bull, fertility marker, frozen-thawed sperm quality, proAKAP4, protein

## Abstract

The precursor protein of A-kinase anchor protein 4 (proAKAP4), which is abundant in the fibrous sheath of sperm, plays an essential role in sperm motility and has been developed into one of the candidate molecular-based fertility markers. This study aimed to identify proAKAP4 in the sperm of Indonesian dairy bulls and to investigate its correlation with various sperm quality characteristics. The findings are expected to be applied and developed to optimize the use of superior dairy bulls. Frozen semen from 20 Indonesian dairy bulls were used, which had previously been classified based on their fertility levels into two groups: high fertility (HF) and low fertility (LF). Analysis of frozen-thawed sperm quality, such as progressive and total motility, sperm kinematics, membrane integrity, mitochondrial membrane potential, acrosome integrity, and capacitation status, was carried out in both groups. ProAKAP4 protein in frozen-thawed sperm was analyzed using the Bull 4MID Kit with the ELISA technique. Each dairy bull showed varying proAKAP4 abundance (*p* < 0.05) from one another. Frozen-thawed sperm quality in HF bulls was higher (*p* < 0.05) than in LF bulls, especially in total and progressive motility, sperm kinematics, membrane integrity, mitochondrial membrane potential, acrosome integrity, and noncapacitated sperm. The parameters of capacitated and acrosome-reacted sperm showed the opposite results, where the quality in LF bulls was higher (*p* < 0.05) than in HF bulls. The results of proAKAP4 identification showed that the protein abundance in HF bulls was higher (*p* < 0.05) than in LF bulls. Correlation analysis confirmed a strong association (*p* < 0.05) between proAKAP4 levels and sperm fertility rate. The proAKAP4 protein has great potential to be developed and applied as a marker for determining the fertility of dairy bulls and for selecting superior dairy bulls.

## 1. Introduction

Bull fertility is chiefly dictated by the capacity of spermatozoa to fertilize ova, resulting in the formation of zygotes [1]. The status or level of bull fertility greatly influences reproductive efficiency, which is crucial for effective and efficient livestock production [2]. The application of artificial insemination (AI) reproductive technology uses frozen-thawed sperm from superior bulls, whose sperm cells can survive during the cryopreservation process and affect the fertility rate [3]. The quality of frozen-thawed sperm used in AI might profoundly influence the fertility rate as the semen from a single bull influences the fertility and production of numerous cows [4]. The selection of superior bulls with good frozen-thawed sperm quality is critical in supporting AI's success in increasing beef and dairy cattle's livestock populations [3].

The increase in the cattle population in Indonesia, especially for dairy cattle, aims to balance the increasing national demand for cow's milk and is inversely proportional to the national cow's milk production, which is still relatively low [5]. Indonesia uses Holstein Friesian breed cattle as the primary source of national cow's milk production, bred with AI technology using frozen-thawed sperm of superior bulls from various AI centers in Indonesia. The dairy bulls used for AI have undergone the breeding soundness examination (BSE) selection process, phenotypically (physical examination and external reproductive organs), libido observation, and standardized sperm quality characteristics [2]. However, the approach through a series of selections still needs to be considered to interpret the fertility level. In addition to the cryopreservation process factor, which can reduce sperm quality after thawing and have an impact on the possibility of affecting fertility rate, recent research has found that sperm quality standards obtained from a series of conventional quality assessments are considered less than optimal in achieving fertility levels in the field [4, 6]. Recent studies have shown that frozen-thawed sperm from dairy bulls used for AI have met the eligibility standards for semen distribution, namely progressive sperm motility after thawing of at least 40%, but have not produced optimal AI success [6–8]. As many as three of six dairy bulls were found to have a fertility rate of only < 30% [9]. Pardede et al. [8] also found that there are still bulls in Indonesia with relatively low fertility (LF) rates (< 50%). The semen quality evaluation method at AI centers in Indonesia is generally still carried out conventionally, including macroscopic and microscopic examinations. This is considered not accurate enough to interpret the fertility of bulls [10–12]. For this reason, a more precise analysis is needed, namely through a molecular analysis approach [1, 13–15].

The presence of specific RNA molecules, proteins, and functional genes through transcriptomic, proteomic, and genomic studies in sperm that play a role in sperm–oocyte function can affect sperm fertility [13–15]. Specific functional molecules can influence several prerequisites that support the reproductive process, such as sperm motility, capacitation, cell protection, acrosome reaction, egg activation, fertilization, and embryo development. RNA molecules and fertility proteins in sperm can be used as potential molecular markers of male reproductive status [1, 13–15]. Pardede et al. [8] found that transition nuclear proteins (TNPs) can be used as sperm motility markers and correlate with bull fertility. A-kinase anchor protein 4 (AKAP4) is a novel molecular marker extensively investigated in various species [16, 17].

Within the fibrous sperm sheath—the focal point of the flagellum in all animals, including bovines—AKAP4 protein is functionally the greatest of numerous essential molecules [16, 18, 19]. AKAP4 is produced as an initial protein known as proAKAP4, a reliable indicator of sperm quality and fertility [20, 21]. AKAP4 in human sperm influences sterile individuals having deficient sperm motility [22]. The concentration of proAKAP4 is associated with total and progressive sperm motility in bulls [16]. Prior research indicates that assessing proAKAP4 levels yields essential insights for forecasting bull fertility [16, 21]. The application of proAKAP4 molecular indicators to measure bull fertility and semen quality is regarded as more efficient, precise, and objective [16, 20]. This study aims to prove the efficacy of the proAKAP4 molecular marker for measuring sperm quality and fertility in frozen-thawed sperm of dairy bulls. The findings of this study are expected to act as a starting point or standard for the future application of protein-based molecular markers for measuring semen quality and fertility in bulls, particularly for dairy cows in Indonesia.

## 2. Materials and Methods

### 2.1. Ethical Approval and Informed Consent

This study only used commercial frozen semen obtained from the AI Center Indonesia and was not directly involved in any production process or with the bulls. The commercial frozen semen was produced from semen collected from 20 dairy bulls (Indonesian Holstein Friesian) owned by the AI Center Indonesia aged 5 to 7 years. Every stage, from storing fresh semen to becoming frozen semen, including the management of bull, followed the operational standards of the AI Center Indonesia, namely SNI ISO 9001: 2015 No. G.01-ID0139-VIII-2019 and SNI ISO 9001: 2015 No. 824 100 16072, supervised by a veterinarian, meeting every animal welfare principle.

### 2.2. Experimental Design

Secondary data from all dairy bulls in this study were collected to obtain the history of frozen semen use for AI programs in the field over the past 2 years. The secondary data were obtained from the Indonesian-Integrated National Animal Health Information System (iSIKHNAS). The data obtained from that system were AI program success data and pregnancy examination results for each inseminated cow, which were then accumulated and further analyzed. More than 10,000 AI success data for the past two years on all bulls used in the study were analyzed for their conception rate percentage according to the method of Pardede et al. [6]. The conception rate was defined as the percentage of pregnant cows (80–100 days postinsemination) among all inseminated cows. The results of this analysis were then used to predict the %fertility rate for each bull in the study. With a range of 50.07%–78.0%, the average fertility rate is 63.04%. Based on their average fertility score and population norm variations, bulls are classified as either LF or high fertility (HF). Bulls were classified as HF if their fertility was above average and LF if their fertility was below normal. Significantly, HF bulls (75.36 ± 0.27%) showed a higher %fertility rate (*p* < 0.05) than LF bulls (50.71 ± 0.08%). For future analysis, the data will be grouped according to whether the bulls are HF or LF (Table 1).

### 2.3. Frozen-Thawed Sperm Quality Assessment

A total of one hundred straws of frozen semen, consisting of five straws per bull from twenty dairy bulls, were used to examine sperm motility, kinematics, and the integrity of the sperm plasma membrane. The motility and kinematic characteristics of sperm cells were assessed using computer-assisted sperm analyses (CASAs; Sperm Vision System, Minitüb, Tiefenbach, Germany). The frozen semen underwent a thawing process for 30 s in a water bath maintained at 37°C. The semen samples underwent dilution in freshly prepared microtubes, adhering to the manufacturer's guidelines, with a precise ratio of 10 μL of semen to 80 μL of dilution media. A 10 μL aliquot of the diluted material was meticulously positioned on a microscope slide and subsequently covered with a cover slip. Two hundred fifty spermatozoa over four fields were assessed using the manufacturer's parameters for bovine semen, using a Carl Zeiss Micro Imaging GmbH (Göttingen, Germany) apparatus with a warm stage maintained at 38°C. The examined motility parameters were progressive total motility and sperm kinematics (velocity). The sperm velocities evaluated at this stage included curvilinear velocity (VCL), straight-line velocity (VSL), and average path velocity (VAP) [23].

**Figure 1:**
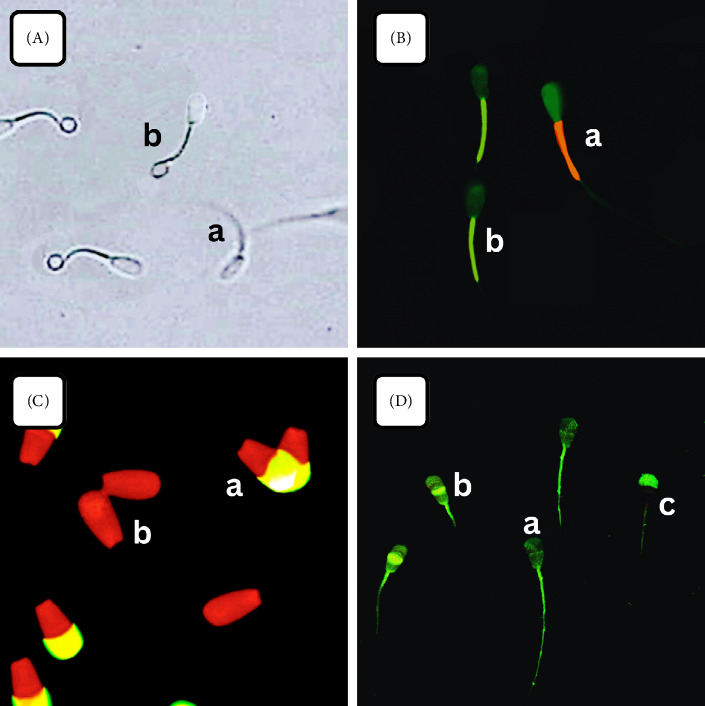
The photomicrograph of sperm in (A) HOS test, (B) JC-1 assay, (C) FITC-PNA and PI-based fluorescent staining, and (D) CTC assay. HOS-negative (absence of coiled tail) is shown in Figure (A-a), and HOS-positive (presence of coiled tail) is shown in Figure (A-b). Sperm show bright yellow/orange in the midpiece if they have high MMP (B-a) and a green reaction if they have low MMP (B-b). Sperm with green fluorescent acrosomes were categorized as intact acrosomes (C-a), whereas sperm without fluorescence were classified as damaged acrosomes (C-b). (D-a) Pattern indicated noncapacitated sperm, the (D-b) pattern indicated capacitated sperm, and the (D-c) pattern indicated acrosome-reacted sperm.

The hypoosmotic swelling (HOS) test was employed to assess the plasma membrane integrity (PMI) of sperm, following the methodology of Rosyada et al. [12]. Semen samples were combined with an HOS test solution and thereafter incubated in a water bath at 37°C for 30 min. Five microliters of each sample was placed onto a glass slide. Each slide was assessed for HOS-positive (indicating the presence of a coiled tail) (Figure 1(A-b)) or HOS-negative (indicating the absence of a coiled tail) (Figure 1(A-a)) sperm by enumerating 250 sperm per sample using a 400× magnification phase-contrast microscope (Figure 1(A)).

One hundred straws of cryopreserved semen (five straws per bull) from twenty dairy bulls were used to assess mitochondrial membrane potential by the JC-1 test, as per Pardede et al. [7]. Semen was tagged with JC-1 (Molecular Probes Inc., Eugene, USA) to assess mitochondrial membrane activity. Semen was centrifuged for 15 min at ambient temperature, after which the sperm pellet was rinsed with phosphate-buffered saline (PBS) (lacking calcium and magnesium; Sigma-Aldrich Chemie GmbH, Steinheim, Germany). The samples underwent centrifugation and were washed twice, after which the sperm pellet was resuspended in 1 mL of PBS. The specimens were stained with 10 μg/mL JC-1 at 37°C for 15 min. Following incubation, the sperm smears were assessed using fluorescence microscopy. A minimum of 250 spermatozoa per sample were evaluated using suitable filters. Sperm exhibited a brilliant yellow/orange hue in the midpiece (Figure 1(B-a)) when mitochondrial activity was strong and a green color response (Figure 1(B-b)) when mitochondrial activity was low or weak (Figure 1(B)).

A total of one hundred straws of frozen semen (five straws per bull) from twenty dairy bulls were used to assess acrosome integrity through the FITC-peanut agglutinin (PNA) and propidium iodide (PI) (Sigma, St. Louis, MO) assay, following the methodology outlined by Hasbi et al. [24]. Frozen semen was thawed in a 37°C water bath for 30 s. A 5 μL of each sample was loaded, smeared onto a slide, and then air-dried at room temperature. The smear samples underwent fixation in 96% ethanol for 10 min at room temperature, followed by air drying. A PNA lectin solution of 30 μL (100 μg/mL) was added and then incubated at 37°C for 30 min. The smear was then dripped with 5 μL (1 μg/μL) of PI solution (Sigma, St. Louis, MO) and incubated for 5 min. After incubation, the smear was washed with PBS and covered using a cover glass. The acrosome integrity status was examined using a fluorescence microscope at 380–420-nm wavelength. Each slide contained an observation of 250 sperm. The examination results were classified into two categories: sperm exhibiting green fluorescent acrosomes (Figure 1(C-a)), identified as having intact acrosomes. Sperm lacking fluorescence (Figure 1(C-b)) were categorized as having damaged acrosomes (Figure 1(C)). Every aspect of staining procedures was conducted in a darkened space.

One hundred straws of frozen semen (five straws per bull) from twenty dairy bulls were used to assess sperm capacitation status through the chlortetracycline (CTC) assay, as described by Satrio et al. [25]. The semen was placed in a microtube for additional examination after being thawed for 30 s in a water bath at 37°C. A confocal laser-scanning microscope (Zeiss LSM 710 META, Germany) operating at 450–490-nm wavelength and with a 520-nm filter barrier was used to analyze 200 sperm samples. Sperm capacitation status was determined using the CTC pattern, which was a colorless sperm head pattern (Figure 1(D-a)) for noncapacitated sperm, a green fluorescent upper half sperm head pattern (Figure 1(D-b)) for capacitated sperm, and a green fluorescent middle part sperm head pattern (Figure 1(D-c)) for acrosome-reacted sperm (Figure 1(D)).

### 2.4. Bovine proAKAP4 Protein Abundance Assessment

The protein abundance of proAKAP4 was assessed using the ELISA method. One hundred twenty frozen semen straws (six straws/bull) from twenty dairy bulls were used. Semen samples were thawed for 30 s in a water bath at 37°C. The semen sample underwent two washes with PBS and was centrifuged at 12,000 g for 15 min. The manufacturer's instructions were to prepare the mixture for ELISA quantification using the Bull 4MID Kit (4BioDx, Lille, France) [16–18, 20–23] after mixing 50 μL of thawed semen samples with 450 μL of Bull Lysis Buffer. Each well of the plate coated with anti-proAKAP4 antibody received 100 μL of lysates. A second horseradish-conjugated proAKAP4 antibody was used to finalize the sandwich ELISA procedure. The substrate solution was applied after the proper washing, and a stop solution was used to end the staining process. The optical density at 450 nm was evaluated through spectrophotometry, as the intensity of the color is directly related to the quantity of proAKAP4 present in each semen sample. The exact amount of proAKAP4 in bull sperm samples was determined by generating parallel standard curves. The results of the proAKAP4 protein abundance assessment were then compared with the standard proAKAP4 protein abundance level, which refers to Bastan and Ackay [26] and Sergeant et al. [27]. ProAKAP4 as a marker of sperm fertility in bulls has four categories, namely poor (< 15 ng/10 M spz), good (15–40 ng/10 M spz), very good (40–60 ng/10 M spz), and very good (> 60 ng/10 M spz).

### 2.5. Statistical Analysis

Twenty dairy bulls were employed for the statistical analysis. The fertility trait data were analyzed through a generalized linear mixed model. The previous subsection detailed the data analysis conducted to categorize dairy bulls according to their fertility rate phenotype. The normality assessment of the study data was conducted using the Shapiro–Wilk test, followed by an evaluation of homogeneity by the Levene test. The study data are regularly distributed and exhibit homogeneity of variance. Data comparing the abundance of proAKAP4 proteins in each bull were analyzed using variance analysis. Duncan's multiple range tests were used to test whether a significant difference was found. The independent *t*-test was used to analyze the difference between the average fertility of the total bull population, the frozen-thawed sperm quality, the fertility rate, and the abundance of proAKAP4 protein. The study's findings are presented as a mean ± standard error. Pearson's test was used to examine the relationship among the different parameters. A scatter plot linearity test revealed a correlation among proAKAP4 protein levels, frozen-thawed sperm quality, and fertility percentage. The data were processed using SPSS version 25.0.

## 3. Results

**Figure 2:**
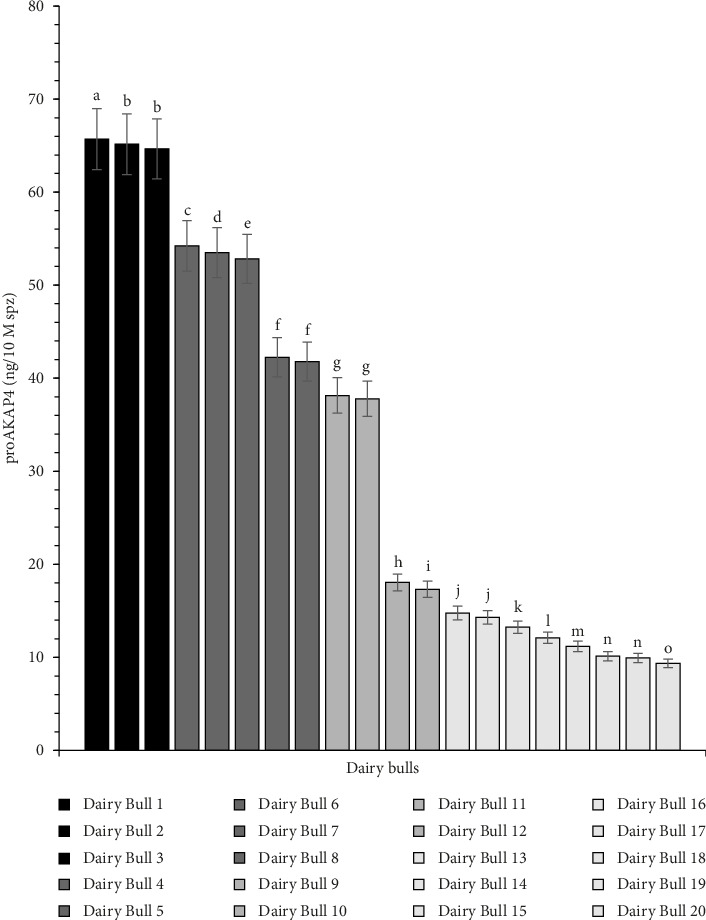
The abundance of proAKAP4 protein in the sperm of each dairy bull used in the study. Different patterns show the categorization of bulls based on proAKAP4 concentration (*p* < 0.05) [26, 27]. Bulls 1–3 were categorized as excellent, Bulls 4–8 were classified as very good, Bulls 9–12 were categorized as good, and Bulls 13–20 were categorized as poor.

The findings of determining the proAKAP4 protein in frozen-thawed sperm from each dairy bull in this research exhibited diverse outcomes. They were significantly different (*p* < 0.05) from one another. According to the various amounts of proAKAP4 protein present in each individual, the dairy bulls in this research can be categorized into four levels of quality: excellent, very good, good, and poor (Figure 2).

Frozen-thawed sperm quality in HF bulls was overall higher (*p* < 0.05) than in LF bulls, especially in total and progressive motility, sperm kinematics (velocity), membrane integrity, mitochondrial membrane potential, acrosome integrity, and noncapacitated sperm. At the same time, the parameters of capacitated and acrosome-reacted sperm showed the opposite results, where the quality in LF bulls was higher (*p* < 0.05) than in HF bulls (Table 2).

**Figure 3:**
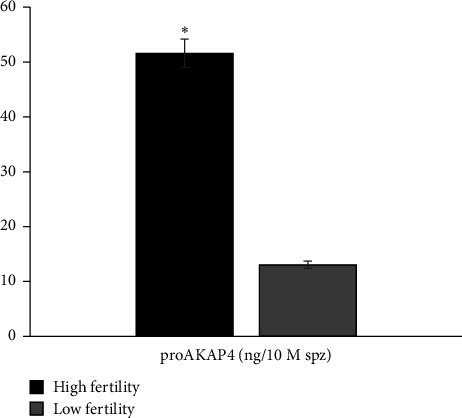
ProAKAP4 protein abundance in frozen-thawed sperm with different fertility groups (HF vs. LF). ⁣^∗^Significant difference when compared to LF (*p* < 0.05).

Furthermore, the results of the identification of proAKAP4 protein abundance also showed that protein abundance in HF bulls was higher (*p* < 0.05) than in LF bulls (Figure 3).

**Figure 4:**
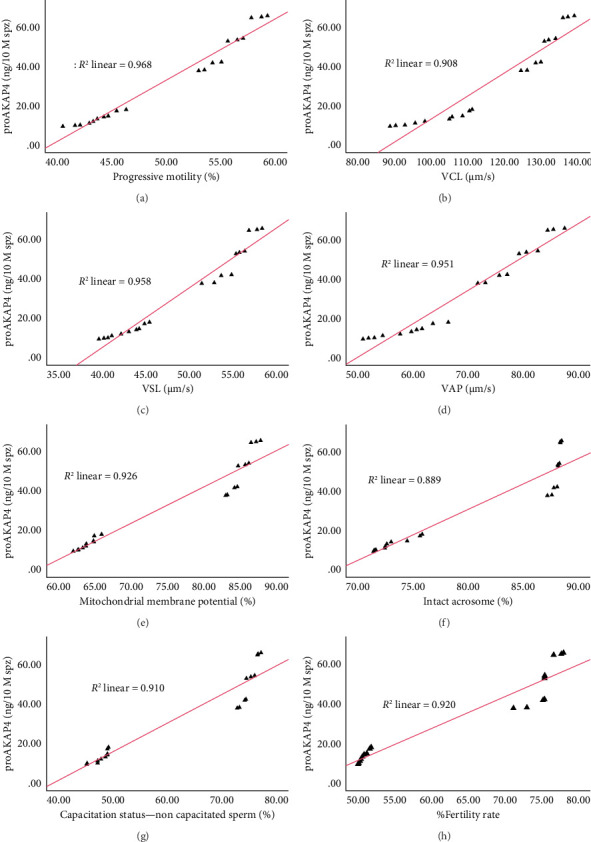
The relationship pattern between the proAKAP4 protein abundance with (a) progressive motility, (b) VCL, (c) VSL, (d) VAP, (e) mitochondrial membrane potential, (f) intact acrosome, (g) capacitation status (noncapacitated sperm), and (h) fertility rate regardless of bull grouping based on fertility level.

The correlation test in this study revealed a strong association (*p* < 0.05) between the abundance of proAKAP4 protein in sperm and frozen-thawed sperm quality (Table 3). The more abundant the proAKAP4 content in sperm, the better the sperm quality, especially in progressive parameters, sperm kinematics (velocity), membrane integrity, mitochondrial membrane potential, acrosome integrity, and noncapacitated sperm. The direct correlation that was proportionally related was further emphasized in the results of this study, as shown in Figure 4. The linear relationship was found between the proAKAP4 protein abundance with progressive motility (Figure 4(a)), VCL (Figure 4(b)), VSL (Figure 4(c)), VAP (Figure 4(d)), mitochondrial membrane potential (Figure 4(e)), intact acrosome (Figure 4(f)), and capacitation status (noncapacitated sperm) (Figure 4(g)). In the results of this study, it was found that proAKAP4 protein is strongly associated and shows a high level of correlation with fertility rate in dairy bulls, with statistical significance (*p* < 0.01) (Table 3 and Figure 4(h)).

## 4. Discussion

The precursor protein of proAKAP4 has been developed as a marker for sperm fertility in various species, including bovine species. Based on the abundance of proAKAP4 protein, dairy bulls in this study can be categorized into each of the above classifications. Bulls 1–3 are classified as excellent, Bulls 4–8 are categorized as very good, Bulls 9–12 are categorized as good, and Bulls 13–20 are categorized as poor. Referring to the findings, it turns out that dairy bulls usually used for AI programs believed to have met specific quality standards are still found to have 8 of 20 dairy bulls classified as poor bulls.

The results of individual proAKAP4 quantification may have a slight impact and differ from the interpretation of group proAKAP4 quantification, HF, and LF. Based on the grouping, half of the dairy bull population is classified as poor bulls. However, based on individual variation, only eight of the total dairy bull population are classified as poor bulls. Further studies may be needed regarding this, or if it will be applied in the selection of superior bulls for dairy bulls, at least it is necessary to limit the category of bulls that will be used as superior bulls for the AI program. Considering that there are four categories of bulls based on their proAKAP4 content, based on this finding, because later quantification will be carried out per individual, bulls with very good and excellent categories will only be used for superior bulls to obtain maximum AI success. However, one thing that can be stated as the main finding in this study, based on individuals or groups, is that proAKAP4 is significantly related to fertility rates.

Poor bulls, categorized based on the abundance of proAKAP4 protein, also show a level of bull fertility that is classified as LF. It is undeniable that poor sperm fertility based on the abundance of proAKAP4 may have an impact on fertilization failure, thereby reducing fertility. However, sperm quality contributes to the percentage of fertilization success in all species, including bovine species. In the AI program, good frozen semen, to optimize fertilization success, must contain good quality in all aspects of sperm quality parameters to maximize fertilization success [28]. A higher number of live, motile, and progressive sperm with intact membranes and acrosomes correlate with an increased likelihood of successful fertilization and egg penetration [29].

Sperm motility is the essential characteristic related to its relationship with fertilization ability and is one of the quality controls of the insemination dose before and after thawing [30, 31]. Aghazarian et al. [32] showed that the integration of motility % and kinematic parameters, particularly sperm velocity (VCL, VAP, and VSL), is significantly associated with fertility. Sperm velocity is a sperm kinematic parameter and part of sperm motility. Adenosine triphosphate (ATP) is necessary for the cell's mitochondria to execute its standard duties, particularly in arriving at the fertilization location within the female reproductive tract [32–34].

Damage to the mitochondrial membrane that disrupts the production of ATP, a source of energy for sperm to move, is also reported as a cause of decreased sperm motility [34, 35]. The mitochondria in sperm consist of helices situated in the midpiece, which generate high-energy ATP via oxidative phosphorylation, supplying energy for sperm movement [34, 36]. In addition to mitochondria, the integrity of the sperm plasma membrane is essential in frozen-thawed sperm, not only because membrane integrity is a prerequisite for the survival of sperm cells but also because unsaturated fatty acids, which are abundant in the sperm plasma membrane, are needed for sperm motility and play a role in membrane fusion during fertilization [37]. Therefore, it is not surprising that the motility, sperm kinematics, membrane integrity, and mitochondrial membrane potential in LF bulls in this research finding are of lower quality than in HF bulls.

In addition to playing a role in membrane fusion during fertilization, the sperm plasma membrane also protects cell organelles, especially the sperm acrosome [38]. The acrosome's function is essential for proper fertilization [39]. The sperm acrosome comprises an inner acrosome membrane and an outer acrosome membrane, which release enzymes for oocyte penetration during the acrosome reaction in fertilization [40]. The higher the sperm with an intact acrosome, the higher the chance of the sperm penetrating the zona pellucida and fusing with the oocyte plasma membrane [41]. This is also represented in the findings of this research, where HF bulls have a higher percentage of intact acrosomes than LF bulls. The chance of successful fertilization in an AI program is higher in bulls with a high percentage of sperm acrosomes.

Semen from bulls for the AI program tends to experience cryodamage due to the cryopreservation process, which impacts decreasing sperm quality [3]. One of the visible impacts of cryodamage is a change in sperm function, including a high incidence of early capacitation and acrosome reaction [42]. Both phenomena are unavoidable in frozen-thawed sperm, as found in this study. The results of this study indicate that capacitation and early acrosome reaction in LF bulls are higher than in HF bulls. The condition of capacitation and early acrosome reaction in sperm due to cryodamage is caused by the disruption of membrane permeability, which results in uncontrolled calcium influx [43]. Sperm capacitation is the main requirement for mammalian sperm to be able to fertilize oocytes [44]. However, when capacitation occurs faster, it can cause sperm to lose their vitality before reaching the fertilization site, or their fertilization ability is low [44]. Therefore, it makes sense that the findings in this study show that noncapacitated sperm in LF bulls is higher than in HF bulls. However, each parameter of sperm quality analyzed in this study is closely correlated with the level of fertility, meaning that good sperm will contribute to the achievement of successful fertilization or AI programs in the field.

However, suppose we only refer to the standard use of frozen semen for the AI program, namely a minimum % progressive motility of 40% [4, 6]. In that case, the fertility rate in the field will not be achieved optimally, as found in this research, where the fertility rate is still only 50% and is still far from the optimal fertility rate of 70%. As one of the proteins contained in the fibrous sheath and playing a role in the function of sperm movement, proAKAP4 in this study showed abundant concentrations in HF bulls. Elevated levels of proAKAP4 signify effective spermatogenesis, leading to a greater quantity of mature sperm with a reduced incidence of tail abnormalities, enhancing sperm motility toward the site of fertilization [17]. ProAKAP4 is a fibrous sperm protein associated with the action of cyclic adenosine monophosphate (cAMP) [16, 18]. cAMP interacts with sperm motility, particularly with the regulatory subunit of protein A kinase, facilitating protein phosphorylation [45]. Protein kinase A can also phosphorylate particular proteins that bind to the DNA promoter region, leading to enhanced transcription [46].

Previous reports indicate that AKAP4 and proAKAP4 are the primary molecules governing the orientation of sperm motility [16–18, 20–22]. AKAP4, or fibrous sheath component 1, is connected to the flagellum [47]. The sperm flagellum's structure constitutes most of the tail, beginning at the neck annulus, which houses the mitochondrial organelles [48]. The flagellum axoneme consists of nine sets of microtubules, namely tubulin A and tubulin B, arranged in a cylindrical shape and firmly attached to the central microtubule pair [49]. AKAP4 is the fibrous sheath's main protein surrounding the microtubule pair [50]—the AKAP4 gene's effect results in sperm tail breakage and unsteady motility [51, 52]. However, the current study shows findings similar to the hypothesis of previous studies. The abundance of proAKAP4 protein is closely correlated with sperm movement components, including motility, velocity parameters (sperm movement patterns), and mitochondria, which are organelles in the sperm flagella that play an active role in producing energy for sperm movement. Furthermore, the relationship between proAKAP4 protein abundance and sperm kinematic parameters, especially sperm velocity parameters (VAP, VCL, and VSL), as found in the current study, has also been previously reported in dog sperm [27, 53].

As a protein localized in the primary piece under basal circumstances and in the principal piece, midpiece, and postacrosomal area [54], it is not surprising that AKAP4 or proAKAP4 plays a role in sperm function, especially those related to the acrosome, as also found in this study. However, the correlation between proAKAP4 protein and sperm quality parameters, especially membrane function and capacitation, has been previously reported. In particular, the correlation between proAKAP4 protein and intact acrosome and several capacitation parameter states, as in this study in dairy bull sperm, has only been reported in recent research. The abundance of proAKAP4 protein in sperm is assumed to be related to the high integrity of the sperm acrosome and noncapacitated sperm to avoid acrosome reactions and early capacitation that result in low sperm fertilization power, resulting in LF. As reported in various studies, proAKAP4 is mainly distributed in the flagella, which plays the most crucial role in the function of sperm movement. Considering that sperm motility or movement is one of the most critical factors sperm needs to reach the fertilization site [16, 20], it is very reasonable if proAKAP4 is considered a potential sperm motility marker.

Furthermore, there is a close correlation between motility and fertility, as well as proAKAP4 with fertility; it is also very possible that this protein can be a potential marker that can be used to assess the fertility level of bulls, especially dairy bulls, as found in this research. However, in addition to the many previous studies stating that the progressive motility parameter is used as a standard for sperm quality for AI programs, this study has again strengthened the hypothesis that conventional sperm quality testing cannot stand alone in distinguishing bull fertility. Conventional sperm quality testing and molecular-based assessment using specific markers, both protein-based as in this research (proAKAP4) and at other molecular levels such as RNA, metabolites, and others, will significantly impact the success of more optimal AI. This will undoubtedly impact increasing reproductive efficiency and economic growth in the dairy cattle farming sector.

Other molecular markers, based on protein, RNA, metabolome, or others such as protamine 1 (PRM1) [6, 11, 12, 15], heat shock protein 70 (HSP70) [7, 10, 12], TNP [8], and others [13], although found to be closely correlated with fertility rate and also various other sperm quality parameters, have not been declared as a product to determine bull fertility, unlike proAKAP4, which has been declared as a commercial product in a protein-based kit that is equipped with specific standards that can classify bulls into several categories. Even referring to the results of this research, proAKAP4 can be applied and used to distinguish various levels of dairy bull fertility. However, when those molecules in sperm [6–8, 10–13, 15] have been further developed to have specific standards to classify bull fertility, further study and engaging discussion will be needed to determine which fertility marker is best for AI programs in the breeding industry, but for now, if you want to use molecular markers, proAKAP4 is by far the best choice and the most ready to use.

## 5. Conclusion

The abundance of proAKAP4 protein in sperm varies between individual dairy bulls and classifies dairy bulls into poor, good, very good, and excellent categories. ProAKAP4 protein, total and progressive motility, sperm kinematics (velocity), membrane integrity, mitochondrial membrane potential, acrosome integrity, and noncapacitated sperm were higher in HF bulls compared with LF bulls. The proAKAP4 protein is associated with several factors of frozen-thawed sperm quality and fertility rates in practical applications. The abundance of proAKAP4 protein is directly proportional (high linearity) to high sperm quality and fertility levels. Furthermore, based on the results obtained, the application of proAKAP4 as one of the advanced fertility assessments can be submitted to policymakers of the AI program to be used as one of the assessment aspects in the selection of dairy bulls in Indonesia. Although in its application, there may be other tests to ensure the strength of this marker in fertility assessment, at least the first step is to combine the use of proAKAP4 with the assessment procedures that have been used so far will be better. When the use of proAKAP4 as a fertility marker is more stable and is considered strong enough to be a single assessment in determining fertility, some of the fertility assessment procedures so far can be reduced one by one to reduce existing operational costs. However, it is undeniable that operational costs in the livestock breeding industry are also a consideration when there are additional procedures in the selection process for superior bulls. Even so, because proAKAP4 is based on a protein quantified using ELISA, which allows for many samples in one test, this may be one of the best and most economical choices.

## Figures and Tables

**Table 1:** Classification of dairy bulls based on %fertility rate was used for further analysis: Bulls 1–10 were categorized as high fertility (HF), and Bulls 11–20 were classified as low fertility (LF).

Bull no.	Fertility status	Average fertility rate (%)	Difference from population average (%)
1	High fertility	78.01	14.98
2		77.72	14.68
3		76.69	13.65
4		75.47	12.43
5		75.46	12.43
6		75.43	12.39
7		75.43	12.39
8		75.22	12.19
9		73.04	10.00
10		71.19	8.15

11	Low fertility	51.80	−11.24
12		51.59	−11.45
13		51.20	−11.84
14		50.84	−12.20
15		50.60	−12.44
16		50.43	−12.60
17		50.31	−12.72
18		50.14	−12.89
19		50.08	−12.96
20		50.07	−12.97

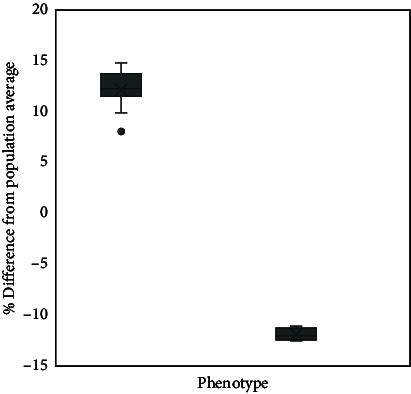			

*Note:* The box plot graph showed a significant difference (*p* < 0.05) on the difference between the average fertility of the total bull population based on phenotype data, high fertility (HF/12.33 ± 0.68), and low fertility (LF/-12.33 ± 0.88) of dairy bulls.

**Table 2:** The difference in the frozen-thawed sperm quality parameters, with fertility groups, HF vs. LF bulls.

Sperm parameters	HF	LF
Total motility (%)	63.17 ± 0.24⁣^∗^	51.61 ± 0.33
Progressive motility (%)	56.07 ± 0.30⁣^∗^	43.48 ± 0.24
Sperm kinematic		
VCL (μm/s)	131.83 ± 0.65⁣^∗^	100.61 ± 1.16⁣^∗^
VAP (μm/s)	79.76 ± 0.73⁣^∗^	57.95 ± 0.72⁣^∗^
VSL (μm/s)	55.31 ± 0.30⁣^∗^	42.51 ± 0.28⁣^∗^
Membrane integrity (%)	79.85 ± 0.60⁣^∗^	61.69 ± 0.38
Mitochondrial membrane potential (%)	85.31 ± 0.37⁣^∗^	63.85 ± 0.28
Acrosome integrity (%)	88.07 ± 0.16⁣^∗^	73.08 ± 0.38
Capacitation status (%)		
Noncapacitated sperm	75.07 ± 0.34⁣^∗^	47.71 ± 0.31
Capacitated sperm	12.31 ± 0.18⁣^∗^	31.19 ± 0.15
Acrosome-reacted sperm	12.62 ± 0.37⁣^∗^	21.09 ± 0.24

Abbreviations: HF, high fertility; LF, low fertility.

⁣^∗^Significant difference when compared to HF (*p* < 0.05).

**Table 3:** Correlation between proAKAP4 protein abundance, semen quality parameters, and fertility rate in the dairy bulls^a^.

Parameters	proAKAP4	%Fertility	PM	TM	VCL	VAP	VSL	MI	MMP	AI	NCS	CS	ARS
proAKAP4	1	0.959	0.967	0.981	0.952	0.973	0.976	0.987	0.948	0.919	0.946	−0.919	−0.914
% Fertility		1	0.963	0.977	0.945	0.939	0.975	0.962	0.983	0.966	0.989	−0.986	−0.905
PM			1	0.990	0.993	0.987	0.992	0.986	0.960	0.949	0.960	−0.943	−0.908
TM				1	0.981	0.984	0.993	0.990	0.970	0.955	0.971	−0.953	−0.919
VCL					1	0.988	0.986	0.978	0.945	0.939	0.946	−0.925	−0.901
VAP						1	0.986	0.989	0.935	0.922	0.934	−0.906	−0.906
VSL							1	0.989	0.967	0.955	0.970	−0.951	−0.921
MI								1	0.953	0.935	0.953	−0.927	−0.917
MMP									1	0.952	0.988	−0.974	−0.924
AI										1	0.962	−0.959	−0.880
NCS											1	−0.985	−0.939
CS												1	−0.985
ARS													1

Abbreviations: AI, acrosome integrity; ARS, acrosome-reacted sperm; CS, capacitated sperm; MI, membrane integrity; MMP, mitochondrial membrane potential; NCS, noncapacitated sperm; PM, progressive motility; TM, total motility; VAP, average path velocity; VCL, curvilinear velocity; VSL, straight-line velocity.

^a^Pearson's correlation represents all values presented, regardless of the grouping of bulls by fertility level. All variables tested were significantly correlated at the 0.01 level.

## Data Availability

The data supporting this study's findings are available from the corresponding author upon reasonable request.
